# Biomaterials-Induced Stem Cells Specific Differentiation Into Intervertebral Disc Lineage Cells

**DOI:** 10.3389/fbioe.2020.00056

**Published:** 2020-02-07

**Authors:** Yizhong Peng, Donghua Huang, Sheng Liu, Jinye Li, Xiangcheng Qing, Zengwu Shao

**Affiliations:** ^1^Department of Orthopaedics, Union Hospital, Tongji Medical College, Huazhong University of Science and Technology, Wuhan, China; ^2^Musculoskeletal Tumor Center, Department of Orthopedics, The Second Affiliated Hospital of Zhejiang University School of Medicine, Hangzhou, China

**Keywords:** intervertebral disc, biomaterials, stem cells, signaling pathway, differentiation

## Abstract

Stem cell therapy, which promotes stem cells differentiation toward specialized cell types, increases the resident population and production of extracellular matrix, and can be used to achieve intervertebral disc (IVD) repair, has drawn great attention for the development of IVD-regenerating materials. Many materials that have been reported in IVD repair have the ability to promote stem cells differentiation. However, due to the limitations of mechanical properties, immunogenicity and uncontrollable deviations in the induction of stem cells differentiation, there are few materials that can currently be translated into clinical applications. In addition to the favorable mechanical properties and biocompatibility of IVD materials, maintaining stem cells activity in the local niche and increasing the ability of stem cells to differentiate into nucleus pulposus (NP) and annulus fibrosus (AF) cells are the basis for promoting the application of IVD-regenerating materials in clinical practice. The purpose of this review is to summarize IVD-regenerating materials that focus on stem cells strategies, analyze the properties of these materials that affect the differentiation of stem cells into IVD-like cells, and then present the limitations of currently used disc materials in the field of stem cell therapy and future research perspectives.

## Introduction

Intervertebral disc degeneration (IVDD) is one of the most common diseases in the field of spinal surgery. It causes changes in the disc height and mechanics of the spine, leading to a deterioration of the patient quality of life (Elabd et al., 2016). According to clinical data, Conventional treatments for IVDD, including physical therapy, oral non-steroidal anti-inflammatory drugs (NSAIDs), epidural injections (Davison et al., 2019), and surgical decompression, fusion (Geisler et al., 2009), and disk replacement (Phillips et al., 2015), are unsatisfactory due to poor therapeutic effects and unexpected complications.

Recently, an increasing number of studies have been conducted to assess the effects of cell therapy for IVDD (Sakai and Andersson, 2015). Several favorable results have been observed for stem cell therapy of IVDD. Though transplanted mesenchymal stem cells (MSCs) can differentiate into intervertebral disc (IVD) cells and significantly promote extracellular matrix (ECM) synthesis in IVD tissues (Chiang et al., 2019) and improve clinical outcome (Noriega et al., 2017) of IVDD patients as well, there were unexpected findings obtained in some studies. An aggravation of IVDD was reported after the injection of MSCs into NP tissues because of the formation of osteophytes (Vadala et al., 2012). Contradiction occurs in pain relieving of intradiscal injection of stem cells. Autologous MSCs therapy significantly improved pain and disability in patients diagnosed with lumbar disc degeneration with intact anulus fibrosus (Orozco et al., 2011), while hematopoietic precursor stem cells (HSCs) intradiscal injection did not improve patients discogenic low back pain (Haufe and Mork, 2006). Apart from operation strategies, source of stem cells and inclusion and exclusion criteria of patients, these unintended consequences might also be attributed to the uncontrolled multidirectional differentiation (Vadala et al., 2012) and the biological activity (Tibiletti et al., 2014) of MSCs that were injected directly into the IVD niche. Strategies to support exogenous stem cells as they adapt to the adverse features of degenerated IVD tissues and to direct stem cell differentiation into NP-/AF-like cells are key points.

The combination of implanted materials and stem cells may provide an effective strategy for these problems. In addition to promoting cell proliferation, migration and ECM production (Illien-Junger et al., 2016a; Perez-Estenaga et al., 2018; Liu et al., 2019), implanted scaffold systems can also direct MSC differentiation into NP-/AF-like cells. An increasing number of engineered materials, including natural scaffolds (Growney Kalaf et al., 2016), synthetic polymers (Helen and Gough, 2008) and their combination (Li et al., 2012), have been applied in IVD repair. Natural scaffolds are biomaterials assembled from various natural polymers, such as silk (Bhunia et al., 2018), polysaccharides (Nair et al., 2015), collagen (Tsaryk et al., 2015), and hyaluronic acid (HA) (Calderon et al., 2010). Bioactive peptides and growth factors (such as TGF-β3) (Tsaryk et al., 2015) that are present in these bioscaffolds effectively enhance stem cell differentiation into chondrocyte-like cells of the IVD (Calderon et al., 2010; Nair et al., 2015; Bhunia et al., 2018). Synthetic polymers can be fabricated in various forms such as injectable and porous ones based on the desired application. Natural-synthetic scaffolds can be produced by simple physical mixing or chemical coupling which the natural segments endow the biocompatibility and the synthetic segments endow the mechanical strength to the final scaffold (Camci-Unal et al., 2013; Pereira et al., 2014).

In this review, we summarized the recent progress in natural, synthetic and hybrid materials for IVD repair and mainly focused on their properties in directing the differentiation of stem cells into IVD-like cells. Potential factors that contribute to stem cell differentiation are carefully discussed, and the future development of materials for IVD regeneration are also highlighted.

## Strategies in Regenerative Medicine for Intervertebral Disc Repair Using Biomaterials That Induce Ivd Cell-Like Differentiation

Recently, many studies have focused on various kinds of biomaterials that are capable of directionally promoting stem cells differentiation toward the IVD cell phenotype (Table 1).

**TABLE 1:** Classification of biomaterials associated with IVD cells lineage differentiation.

Category	Biomaterials	Crosslinking agent	Stem cells	Differentiation	*In vitro* markers	Signaling pathway	*In vivo* or *ex vivo*	Citation
Natural biomaterials	Collagen type II-chondroitin sulfate hydrogel	Genipin	ADSCs	NPCs	Collagen type II, Acan, Krt19, Pax1, Sox-9	NA	Rat	Zhou et al., 2018c
	Gelatin-hyaluronic acid methacrylate hydrogel	Genipin	ADSCs	NPCs	Collagen type II, Acan, Ovos2, PAX1, Foxf1, CD24, GLUT-1, GPC3, Krt19, MMP-2	NA	Rat	Chen et al., 2019b
	Silk-based multilayered angle-ply scaffold	None	BMSCs	AFCs	Collagen type I, Sox-9, Acan	NA	NA	Bhunia et al., 2018
	Chitosan-hyaluronic acid hydrogel	None	ADSCs	NPCs	Collagen type II, Acan, Krt18, CD24	NA	NA	Zhu et al., 2017b
	Decellularized allogeneic intervertebral disc	None	BMSCs	IVD-like cells	Collagen type II, Collagen type II/I, Acan, Sox-9, GPC3	NA	Rabbit	Lin et al., 2016
	Acellular porcine NP hydrogel	None	ADSCs	NPCs	Collagen type II, Collagen type III, Sox-9, TIMP-1, Pax-1	NA	NA	Mercuri et al., 2013
	NP cell-derived acellular matrix	None	BMSCs	NPCs	Collagen type II, GPC3, Foxf1	NA	Rabbit	Yuan et al., 2013
	Dextran, chitosan, and teleostean combined hydrogel	None	MSCs	NPCs	Acan, Collagen type II, Sox-9	NA	*Ex vivo*	Smith et al., 2014
	Temperature sensitive hydrogel chitosan-glycerophosphate (C/Gp)	None	BMSCs	NPCs	Sox-9, Acan, Collagen type II	NA	NA	Richardson et al., 2008
	Chitosan and alginate gel scaffold	None	ADSCs	NPCs	Acan, Collagen type II, HIF-1α	NA	NA	Zhang et al., 2014
	Alginate and chitosan hydrogels	None	BMSCs	NPCs	Collagen type II	NA	NA	Naqvi and Buckley, 2015
	Self-assembling peptides	None	NPSCs	NPCs	Collagen type II, Acan, Sox-9	NA	*Ex vivo*	Wu et al., 2016
	Spherical niche-like structures of collagen type II and hyaluronan	None	ADSCs	NPCs	Collagen type II, Acan, Sox-9	Rock1/integrin α10 signaling pathway	NA	Fontana et al., 2014
	Collagen type II hydrogels	EDAC/NHS	ADSCs	NPCs	Acan, Collagen type II, Sox-9, Krt19	NA	NA	Zhou et al., 2016
	Collagen type II scaffold	Genipin	ADSCs	NPCs	Acan, Collagen type II, Sox-9, Krt19, Gdf10, Pax1	Shh signaling pathway	NA	Zhou et al., 2018b
	Collagen type II hydrogels	None	ADSCs	NPCs	Acan, Collagen type II, Krt19, Pax1	Shh signaling pathway	Rat	Zhou et al., 2018a
	KLD-12 polypeptide/TGF-β1-nanofiber gel	None	BMSCs	NPCs	Collagen type II, Acan	NA	NA	Bian and Sun, 2015
	dNP-based cell delivery system	None	ADSCs	NPCs	Acan, Collagen type II, Sox9, Krt19, Pax1	NA	Rabbit	Zhou et al., 2018d
Synthetic biomaterials	pNIPAM hydrogel system	None	BMSCs	NPCs	Acan, Collagen type II, CS, HIF1α, PAX1, Foxf1	NA	NA	Thorpe et al., 2016
	Nanofibrous poly(l-lactide; PLLA) scaffolds	None	BMSCs	NPCs	Collagen type II, Sox-9, Acan, HIF1α	NA	Rabbit	Feng et al., 2011
	Heparin-PEAD conjugated vehicle	None	ADSCs	NPCs	Collagen type II, Acan, PAX1, Foxf1	NA	Rat	Zhu et al., 2019
	PECUU materials	None	AFSCs	AFCs	Collagen type I	NA	NA	Zhu et al., 2016
	PECUU materials	None	AFSCs/BMSCs	IVD-like cells	Acan, Collagen type I, Collagen type II	NA	NA	Guo et al., 2015
	PTMC scaffold	None	ADSCs	AFCs	Acan, Collagen type I, Collagen type II	NA	NA	Blanquer et al., 2017
	PLLA and PCL nanofibers	None	BMSCs	AFCs/NPCs	Collagen type I/Collagen type II, Acan, Sox-9	NA	*Ex vivo*	Tsai et al., 2014
	p(HEMA-co-APMA)g PAA hydrogel	None	BMSCs	NPCs	Sox-9, Acan, Collagen type II	NA	*Ex vivo*	Kumar et al., 2016
	PECUU scaffolds	None	AFSCs	AFCs	Collagen type I, Acan	NA	NA	Liu et al., 2015
	PTMC scaffold combined with MSCs and a PU membrane	None	BMSCs	AFCs	Col V, Acan	NA	*Ex vivo*	Pirvu et al., 2015
Biosynthetic biomaterials	T1307-fibrinogen hydrogel	None	NPSCs	NPCs	Sox-9, Acan	NA	NA	Navaro et al., 2015
	HA-pNIPAM hydrogel	None	BMSCs	NPCs	Sox-9, Acan, Krt19, CD24, CA12, Collagen type II	NA	*Ex vivo*	Peroglio et al., 2013
	PEG-HA-PPS hydrogel	None	MSCs	NPCs	Collagen type I, Collagen type II	NA	NA	Frith et al., 2013

*NP, nucleus pulposus; AF, annulus fibrosus; NPCs, nucleus pulposus cells; AFCs, annulus fibrosus cells; NPSCs, nucleus pulposus–derived stem cells; ADSCs, adipose-derived stem cells; BMSCs, bone marrow mesenchymal stem cells; MSCs, mesenchymal stem cells; IVD, intervertebral disc; SDSCs, synovium-derived stem cells; HUCMSCs, Human umbilical cord mesenchymal stromal cells; PEAD, polycation poly(ethylene argininylaspartate diglyceride); PLLA, poly-L-lactic acid; PTMC, poly (trimethylene carbonate); PECUU, poly(ether carbonate urethane)urea; PLCA, poly(lactide-co-glycolide); PLLA, poly-L-lactic acid; PCL, polycaprolactone; p(HEMA-co-APMA)g PAA, polyhydroxyl ethyl methacrylate-co-N-3-aminopropylme thacrylamide grafted with polyamidoamine; RADA-KPSS was manufactured by conjugating a bioactive motif derived from BMP-7 [KPSS] onto the C terminal of RADA16-I; PTMC, poly(trimethylene carbonate); PU, poly(ester-urethane); CP, chitosan-poly(hydroxybutyrate-co-valerate); CS, chondroitin sulfate; HA-pNIPAM, hyaluronan-poly(N-isopropylacrylamide); PPS, pentosan polysulfate; PEG, poly(ethylene glycol); EDAC, N,N-(3-dimethylaminopropyl)-N0-ethyl carbodiimide; NHS, N-hydroxysuccinimide. NA, not available.*

### Natural Biomaterials

Collagen is the most abundant component within IVD tissues. Collagen I and II account for 80% of the total collagen content. Thus, to mimic the natural structure of IVD tissues, collagen I/II are widely used materials for IVD regeneration. However, biomaterials consisting of collagen alone are less efficient in promoting stem cell differentiation toward IVD cells, and supplementation with crosslinking agents of appropriate concentration is more likely to increase adipose-derived stem cells (ADSCs) gene and protein expression of Collagen type II, Sox-9 and aggrecan, suggesting a NP-like phenotype (Zhou et al., 2018b). Gelatine, which is a denatured form of collagen, has been used for NP-like differentiation of ADSCs and IVD repair. Its combination with HA and methacrylate forms a photo crosslinked hydrogel, improves the efficacy of NP-like differentiation (elevated PAX1, CD24, aggrecan, Collagen type II et al.) and also significantly alleviates IVDD *in vivo* (Chen et al., 2019b).

Hydration of NP tissues is essential for maintaining resistance to axial compression and hydrostatic pressure (Schmidt et al., 2016). HA and other glycosaminoglycans (GAGs) are key components that help maintain tissue hydration and improve tissue differentiation-inducing capacity; thus, they are often applied as IVD-regenerating biomaterials. For example, HA combined with platelet-rich plasma and batroxobin (a gelling agent) has been shown to be a novel injectable hydrogel that could serve as a potential cell carrier for IVD regeneration, and MSCs cultured in the gel in a 3D manner were found to produce increased amounts of GAGs without TGF-β1 supplementation (Vadala et al., 2017). Many other biomaterials have included HA as a component and revealed an enhanced capacity for NP cell-like differentiation either *in vivo* or *in vitro* (Calderon et al., 2010; Tsaryk et al., 2015; Zhu et al., 2017b).

**FIGURE 1:**
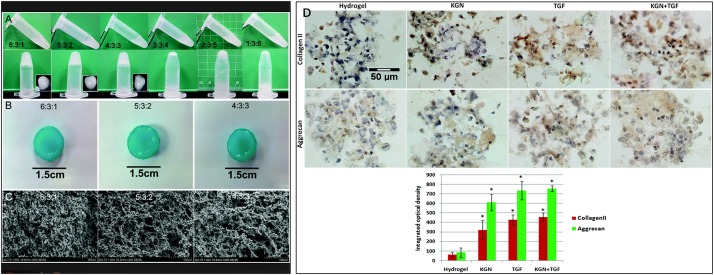
The fabrication and structure of hydrogels. **(A)** Images of CS, GP, and HA solutions before (sol) and after (gel) incubation at 37°C. The 3: 3: 4, 2: 3: 5 and 1: 3: 6 mixtures were unable to form gels, even after an extended incubation time. **(B)** Macroscopic images of CS/HA hydrogels stained with alcian blue after incubation in PBS at 37°C. **(C)** SEM images of hydrogels. The structure of the 4 : 3 : 3 hydrogel was too loose to be broken. The scale bar indicates 100 μm. **(D)** The expression of collagen type II and aggrecan by immunohistochemical staining. Both KGN and TGF-β promoted the differentiation of ADSCs in the hydrogel scaffold to similar extents. A semi-quantitative analysis was performed to confirm the results. The scale bar indicates 50 μm. All data are presented as mean ± SEM. ^∗^ Means significance compared to Hydrogel. Published by The Royal Society of Chemistry (RSC) on behalf of the Centre National de la Recherche Scientifique (CNRS) and the RSC (Zhu et al., 2017b).

Chitosan, which is derived from chitin, is a natural non-sulfated GAG that is widely utilized in various regenerative biomaterials due to its low toxicity, non-immunogenicity, biocompatibility, and intrinsic antibacterial and adhesive properties (Li et al., 2018). However, due to its poor mechanical strength, chitosan is typically combined with other kinds of materials (Xie et al., 2018), such as alginate, gelatine, HA and nanoparticles, to overcome this disadvantage (Naqvi and Buckley, 2015; Teixeira et al., 2016; Zhu et al., 2017b). A kartogenin (KGN)-conjugated chitosan-HA hydrogel has been fabricated (Figures 1A–C) and has achieved controlled release of KGN, which is a chondrogenic and chondroprotective agent, promoting ADSC proliferation and Collagen type II, aggrecan, CD24, Krt18, et al. gene and protein expression (Figure 1D; Zhu et al., 2017b).

**FIGURE 2:**
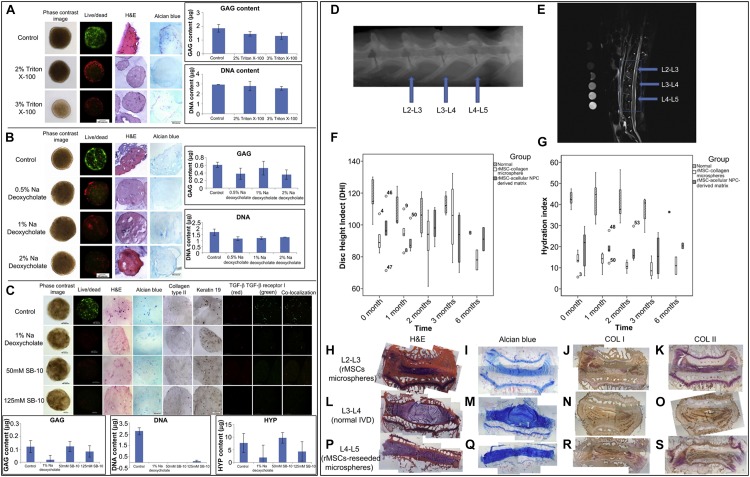
Optimization of the decellularization protocol. **(A)** A comparison of decellularization with Triton X-100 at different concentrations. **(B)** A comparison of decellularization with Na deoxycholate at different concentrations. **(C)** A comparison of decellularization with 1% Na deoxycholate and a combination of 50 or 125 mM SB-10 and 0.6 mM SB-16/0.14% Triton X200. Scale bars: 100 microns; GAG, DNA and HYP assay: *n* = 3. **(D)** A representative X-ray radiograph; **(E)** representative MRI image; **(F)** radiographic analysis of the disc height; and **(G)** water content indicated by MRI. **(H–K)** L2-L3 levels of IVDs (injection of rMSC microspheres). **(L–O)** L3-L4 levels of IVDs (normal). **(P–S)** L4-L5 levels of IVDs (injection of MSC-seeded microspheres). **(H,L,P)** Routine H&E staining of IVDs. **(I,M,Q)** Alcian blue staining of IVDs, **(J,N,R)** Immunohistochemical staining of collagen type I. **(K,O,S)** Immunohistochemical staining of collagen type II; *n* = 2–5 (Yuan et al., 2013). Copyright©2013 Elsevier.

There has been increasing interest in utilizing biological scaffolds composed of ECM from decellularized tissue over the past decade (Saldin et al., 2017). Decellularized ECM retains its native microstructure and biocompatibility and reduces inflammatory and immune responses (Yuan et al., 2013). How to maintain ECM and eliminate cellular components to the greatest extent is a substantial concern in generating decellularized materials (Figures 2A,B). Triton-100, SDS (Yuan et al., 2013) or ethylenediaminetetraacetic acid (EDTA) (Hensley et al., 2018) are widely applied to remove cellular components and are crucial in IVD decellularized scaffolds preparation, and the proper choice of agents concentration and application time are essential to fully remove resident cells while preserving ECM, including collagen, GAGs, proteoglycans and growth factors (Saldin et al., 2017) (another review has fully discussed the efficacy of various decellularization preparation). Differing from cellular materials, immunogenicity of ECM components is generally conservative among species. Therefore, it is well tolerant when used as allografts (Chen et al., 2019a) or xenografts (Schneider et al., 2018). Decellularized IVD scaffolds significantly promoted MSC viability and increased Collagen type II, Collagen type II/type I, AGN, Sox-9, GPC3 expression *in vitro*, indicating a NP-like phenotype differentiation, and alleviated aggravation of disc height decline and microstructure disruption on rabbit model (Lin et al., 2016). A crosslinking agent can significantly enhance the mechanical properties of decellularized NP scaffolds and has been used to fabricate an injectable cell delivery system that induced ADSCs into a NP-like phenotype (elevated Krt19 and Pax1 expression) *in vitro* (Figure 2C) and achieved IVD regeneration in an *in vivo* rabbit model (Zhou et al., 2018d; Figures 2D–S).

Chemical modifications of natural materials are common approaches that provide these materials with novel properties, such as photo-crosslinking and thermo-sensitivity. A photo crosslinked gelatine-HA methacrylate (GelHA) hydrogel retains the differentiation-inducing capacity of HA *in vitro* and contained facile gelatine (Chen et al., 2019b), which is especially practical for IVD repair *in vivo*. In addition, mixing beta-glycerophosphate, which is a weak base, with chitosan has been shown to generate a thermosensitive biomaterial that can be triggered to gel upon heating (Cho et al., 2005), making it possible to distribute stem cells homogeneously within materials at room temperature before gelation to create an injectable stem cell delivery system (Richardson et al., 2008).

### Synthetic Polymers

Although natural biomaterials typically accurately simulate the native microstructure and components that are biocompatible and multifunctional, their physical features and mechanical properties are often limited. Thus, a synthetic matrix that is well composed and possesses controlled biodegradability should be investigated.

Due to its characteristics that enable easy modification, poly (ether carbonate urethane) urea (PECUU) materials are widely applied to fabricate materials with a variety of elastic moduli (Zhu et al., 2016). The expression level of Collagen type I in AF-derived stem cells (AFSCs) cultured on PECUU fibrous scaffolds has been shown to increase as the elasticity of scaffolds increased, while Collagen type II and aggrecan showed different tendencies (Zhu et al., 2016). Moreover, PECUU materials consisting of random or aligned fibers have also been produced (Liu et al., 2015). AFSCs cultured on aligned PECUU scaffold materials were more elongated and exhibited higher expression levels of Collagen type I and aggrecan, indicating an outer AF cell phenotype. For an alternative arrangement of fibers, PECUU materials are a great choice for investigating the effect of mechanical properties on cell differentiation.

Because of the controlled synthetic process and well-designed composition, a synthetic matrix can achieve multifunctional purposes. A poly(trimethylene carbonate) (PTMC) scaffold covered with a poly(ester-urethane) (PU) membrane and seeded with human bone marrow-derived MSCs (hBMSCs) has been developed to provide a complex structure for immediate closure of ruptured AF tissues (Pirvu et al., 2015). This scaffold of the cellular delivery system can withstand dynamic loads, restore disc height of ruptured bovine AF tissues, and increase Collagen type V expression, indicating an *in situ* differentiation capability (Pirvu et al., 2015).

Recently, temperature sensitive materials are most extensively studied because the parameter change naturally and can be easily controlled. Among all these temperature response materials, poly (N-isopropylacrylamide) (pNIPAM) is most promising for biological applications because of its well defined structure and appropriate thermodynamic property. pNIPAM could be prepared into smart hydrogel which is negatively thermosensitive (Rosenzweig et al., 2018). In other words, pNIPAM could contract upon increase in temperature beyond their critical temperature and then forms a gel. The volume phase transition temperature for pNIPAM hydrogel is approximately 34°C, which is a little bit higher than that of the polymer in aqueous solution (about 32°C) (Haq et al., 2017). And the process of solution-gel transition is reversible. A laponite crosslinked pNIPAM-co-DMA was observed to have the elastic modulus (G′) up to 5.5 ± 0.2 × 10^5^ Pa and could effectively restore disc height and could potentially increase NP matrix markers (aggrecan, collagen type II, chondroitin sulfate) as well as NP phenotypic markers (HIF1α, PAX1, FOXF1) of hMSCs (human MSCs) in the absence of growth factors (Thorpe et al., 2016, 2017). What’s more, after crosslinked by laponite, the gelation process became irreversible. In other words, this system was stable because it did not re-liquefy when the temperature increased. Thus, the well fabricated laponite crosslinked pNIPAM-co-DMA might be a potential material for the repair of IVD tissues.

Typically, activated growth factors do not maintain sufficient long-term concentrations in specific regions because of their short elimination half-life (Zhao et al., 2015). Manipulating a synthetic matrix could result in well-controlled chemical components and growth factor supplementation conditions. Heparin is an ideal bridging agent that binds growth factors to retain their bioactivity (Chu et al., 2011). A growth factor delivery vehicle comprised of heparin and the synthetic material poly(ethylene argininylaspartate diglyceride) (PEAD) has been developed and has achieved the controlled release of GDF-5 for at least 4 weeks, which supported the differentiation of ADSCs and restoration of degenerated NP *in vivo* (Zhu et al., 2019).

### Biosynthetic Materials

The controlled biodegradability, favorable physical properties and modified chemical-carrying capacity of synthetic materials make them an ideal choice for tissue engineering and IVD regeneration. However, their biocompatibility and potential cytotoxicity limit their applications. On the other hand, natural materials, including collagen (Zhou et al., 2018c), fibrin (Long et al., 2018), HA (Kazezian et al., 2017), and chitosan (Doench et al., 2019), provide cells with adhesive surfaces and have low cytotoxicity, thus possessing good cellular compatibility. Therefore, the combination of these two types of materials is a potential approach to fabricate bioscaffolds with reliable mechanical properties and biological compatibility (Frith et al., 2013).

Pentosan polysulfate (PPS), which is a synthetic GAG-like factor, significantly promoted mesenchymal precursor cells (MPCs) proliferation at the concentrations of 1 to 5 mg/ml, and induced more proteoglycan biosynthesis, Collagen type I and type II deposition in pellet culture (Ghosh et al., 2010). Due to its biocompatibility and differentiation effects, PPS has been covalently bonded within HA/PEG hydrogels (Frith et al., 2013). HA/PEG-PPS decreased the time consumed for G′ reaching 1 kDa and enhanced the potential for NP cell-like differentiation with decreased Collagen type I deposition, increased Collagen type II expression and GAG contents *in vitro* (Frith et al., 2013, 2014). However, the concentration of PPS (5 μg/ml) reported in these studies hindered cells proliferation, therefore the hydrogels did not performed ideal biocompatibility and need further modification.

Fibrinogen has been conjugated to Tetronic-tetraacrylate (T1307-TA) by a Michael-type addition reaction. By increasing the ratio of Tetronic 1307-TA to fibrinogen, biosynthetic materials with different elastic moduli have been created. Tetronic 1307-TA-fibrinogen with a lower modulus promotes improved cell survival and chondrogenic differentiation of nucleus pulposus–derived stem cells (NPSCs) (Navaro et al., 2015).

As mentioned above, pNIPAM possesses its potential value for biological applications but unable to use directly. Some biomaterials were also mixed into pNIPAM in order to alter its properties. A thermoreversible hyaluronan-based-pNIPAM (HA-pNIPAM) was produced as nucleus pulposus cells (NPC) carrier (Peroglio et al., 2012). With the increased pNIPAM molecular weight of the HA-pNIPAM hydrogen system from 10 to 35 × 10^3^ g/mol, the elastic modulus (G′) strengthened from 0.005 to 16 kPa at 37°C. HA-pNIPAM with the molecular weight pNIPAM of 20 × 10^3^ g mol^–1^ (HA-pNIPAM20) was observed a best biocompatibility and was suitable for NPCs carriers. Another study observed that HA-pNIPAM20 characterized a low viscosity at 20°C, a rapid gelling at 37°C without volume change upon gelling, and G′ was 140 Pa at 37°C, which allows for cells preculture and recollection after gelation, thus making the material a promising cells delivery system (Mortisen et al., 2010). Besides the fine physicochemical properties, the HA-pNIPAM hydrogel also support MSCs differentiation by upregulating disc markers, such as KRT19, CD24 as well as FOXF1 both *in vitro* and *ex vivo* (Peroglio et al., 2013). However, fast thermos-response and simultaneous improvement in mechanical properties are still challenges, especially in biological applications (injectable hydrogel for tissue regenerations et al.) (Haq et al., 2017). Specific designs in combination with mechanical strength, controlled biological degradation and thermosensitive properties, are still required to be addressed carefully.

## Biomaterial Features for Stem Cell Differentiation

**FIGURE 3:**
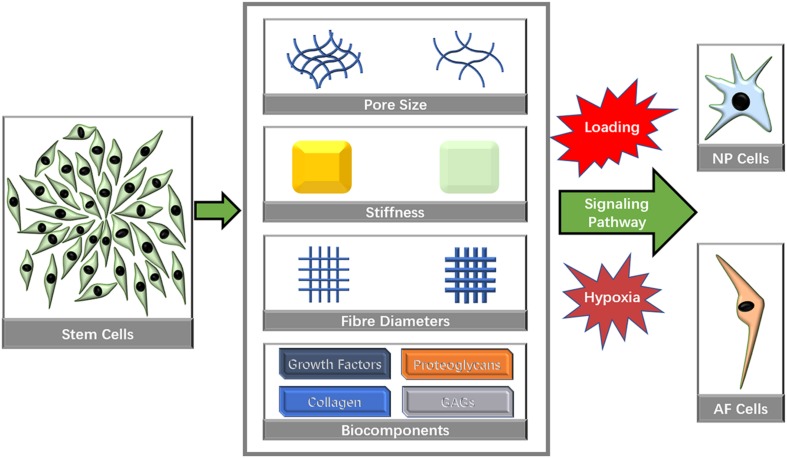
Biomaterial characteristics that influence stem cell directional differentiation toward IVD cells.

Many microstructural characteristics, such as pore size, fiber size and inherent mechanical factors, have been identified to affect the directional differentiation of stem cells (Figure 3).

### Mechanical Characteristics

**FIGURE 4:**
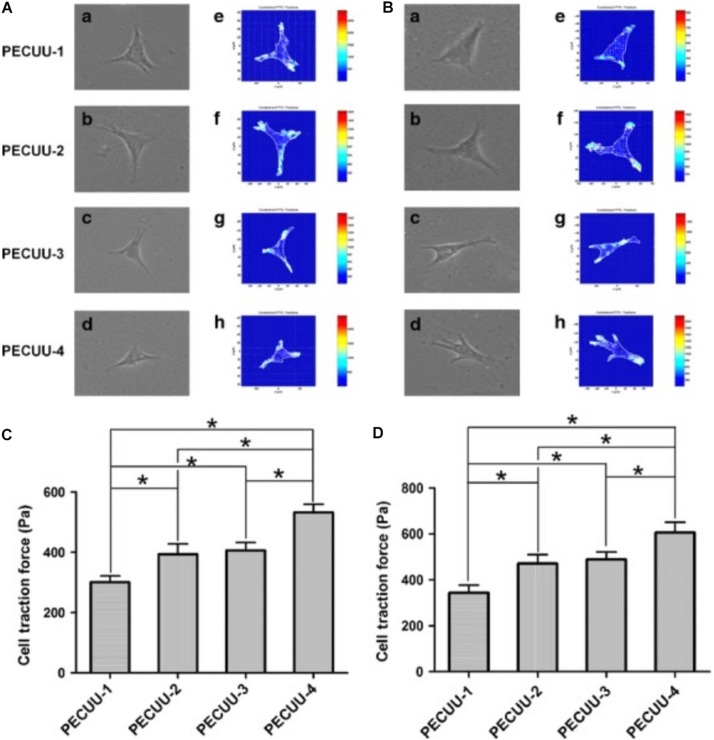
Cell traction force microscopy (CTFM) measurement of AFSCs and BMSCs cultured on poly(ether carbonate urethane)-urea (PECUU) scaffolds of different elastic modulus for 2 weeks. **(A,B)** CTFM for measuring CTFs of AFSCs and tBMSCs, respectively. **(a–d)** Phase contrast images of cells; **(e–h)** CTF maps of corresponding cells. **(C,D)** The computed CTFs of AFSCs and tBMSCs, respectively. All data are presented as mean ± SEM. The symbol “*” indicates significant difference between groups (*p* < 0.05, *n* ≥ 20) (Guo et al., 2015). Copyright©2015 Wiley.

Micromechanical properties determined by the microstructure of a matrix have been identified to affect cell differentiation (Wen et al., 2014; Ye et al., 2015). PECUU materials with a smaller elastic modulus (G′ = 2 MPa) have been shown to exert a greater capacity to induce NP-lineage differentiation, while a larger Young’s modulus (13 MPa) has been shown to markedly increase the Collagen type I expression level (Guo et al., 2015; Zhu et al., 2016). Tetronic 1307-tetraacrylate (T1307-TA) conjugated with fibrinogen with a lower elastic modulus (G′ = 1 kPa) has been shown to promote the differentiation of NPSCs toward a chondrogenic differentiation pathway for IVD regeneration, whereas matrices with a higher modulus (G′ = 2 kPa) have been shown to promote osteogenic differentiation (Navaro et al., 2015). Cell traction forces gradually increased as the stiffness of PECUU substrate is augmented (Figure 4), which is corresponding to the native feature of AF cells from inner AF to outer AF, and may contribute to the effect of stiffness on cellular differentiation (Guo et al., 2015; Zhu et al., 2016). Similarly, early cardiac transcription factors expression is dependent on the cellular response to increasing stiffness, which is also consistent to age related development of the complex ECM (Gershlak et al., 2013), suggesting that the response of cellular traction forces to surrounding ECM is an important factor for tissue specific differentiation. Some studies have emphasized the importance of bionic micromechanical characteristics (Wan et al., 2016). More specifically, biomaterials with natural NP and AF structures have been recommended for IVD reconstruction (Zvicer and Obradovic, 2018). Nevertheless, the complex modulus of the NP is 22 kPa (Bron et al., 2009), and the AF extra-fibrillar matrix modulus ranges from 10 to 50 kPa (Cortes et al., 2013), which is much greater than the modulus that favors IVD cell differentiation. MSCs cultured on polyacrylamide (PA) scaffolds with a G′ of 12 kPa have been shown to generate more F-actin cytoskeletons and bundled stress fibers and were induced into the osteogenic lineage (Hsieh et al., 2016). However, the osteogenesis of stem cells within IVD is associated with endochondral ossification (Illien-Junger et al., 2016b), and its imbalance with adipogenesis induced by LPS could lead to IVDD (Zhu et al., 2017a). Thus, the imbalance between the requirement of natural mechanical strength and efficient stem cells therapy urges the development of hybrid biomaterials that contain at least two components with different strength. One with lower modulus (around 1 kPa) increases the tendency of tissue specific differentiation, while one with nature-matched strength (around 10–50 kPa) can sustain axial compressive force and shear force within IVD and also prevent the leakage of the component with lower modulus.

### Pore Size

The pore size of a scaffold microarchitecture affects cell adhesion, which significantly influences cell interactions, migration, growth and differentiation. Both larger (>200 μm) and smaller (≤200 μm) pore sizes promote cell proliferation (Griffon et al., 2006; Nava et al., 2016; Kim et al., 2017), while larger pore sizes are more favorable for chondrogenesis (Oh et al., 2010).

**FIGURE 5:**
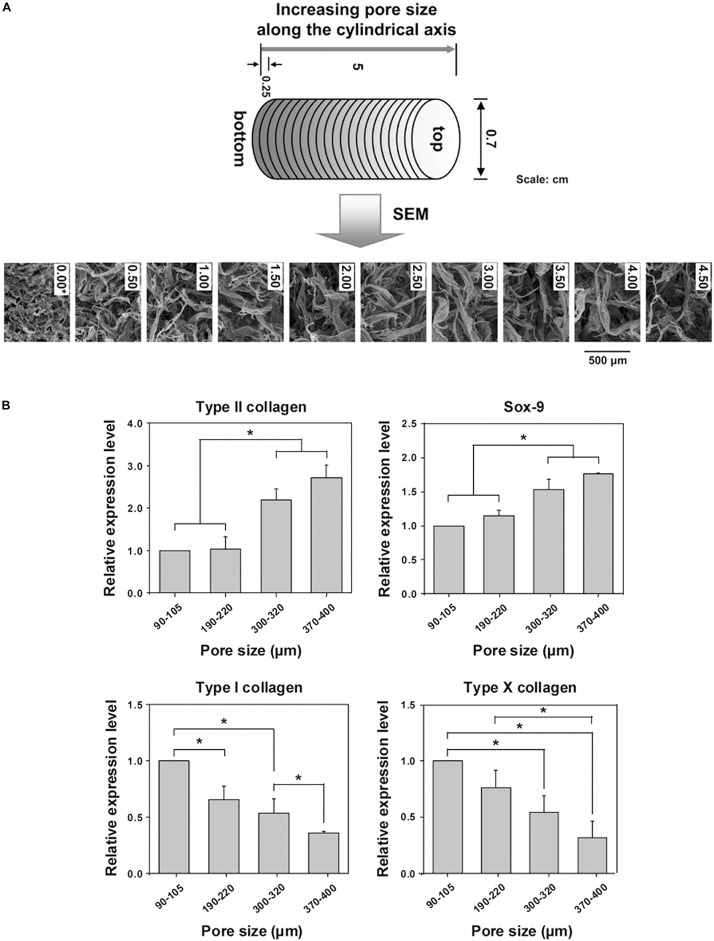
Investigation of pore size effect on differentiation of adipose stem cells using a PCL scaffold. **(A)** SEM photographs of the top surfaces of the selected PCL scaffold sections along the longitudinal direction. **(B)** Real-time PCR after 3 weeks of *in vitro* ADSCs culture in the scaffold sections with different pore size ranges; (*n* = 3; *p < 0.05). The mRNA expressions were measured and normalized against GAPDH (Collagen type II and Sox-9 are positive markers; Collagen type I and X are negative markers for chondrogenic differentiation of ADSCs) (Oh et al., 2010). Reprinted with permission from Oh et al. (2010) Copyright © 2010 American Chemical Society.

A novel integrated biphasic IVD scaffold that was fabricated using a simple freeze-drying and crosslinking technique of pig bone matrix gelatine (BMG) for the outer AF phase with a large pore size of 401.4 ± 13.1 μm and pig acellular cartilage ECM (ACECM) for the inner NP phase with a pore size of 231.6 ± 57.2 μm has been shown to support cell proliferation and maintain the cellular phenotype (Xu et al., 2015). An AF biomimetic structure with a pore size of 343.0 ± 88.25 μm consisting of pig proximal femoral cancellous bone rings has been shown to be an ideal scaffold for ADSC proliferation (Xu et al., 2013). PLGA scaffolds designed for NP regeneration have been fabricated by solvent casting/salt-leaching with pore sizes of 90–180, 180–250, 250–355, and 355–425 μm, and pore sizes of 90–250 μm showed better effects on cell proliferation, while the content of GAG and collagen increased significantly in scaffolds with larger pore size (250–355, 355–425 μm) (Kim et al., 2017). Moreover, porosities above 90% have also been shown to be beneficial for differentiation toward NP-like cells (Zhou et al., 2016). Similarly, polycaprolactone (PCL) (Figure 5A) cylindrical scaffolds with larger pore size (370–400 μm) increased Collagen type II, sox-9, GAG content and GAG/DNA index, while inhibited Collagen type I and X expression in ADSC *in vitro* (Oh et al., 2010; Im et al., 2012; Figure 5B). However, scaffolds made from poly(urethane urea) showed contradictory outcome, in which larger pore size (300–500 μm) downregulated proteoglycan production and Collagen type II expression but promoted Collagen type I expression (Stenhamre et al., 2011; Nava et al., 2016).

The well permeability of nutrients and oxygen of scaffold provides sufficient supply for stem cells and leads to better chondrogenic differentiation (Oh et al., 2010). Apparently, larger pore sizes allow for sufficient cell space, easy nutrient diffusion and efficient discharge of metabolites, which leads to improved cell proliferation and differentiation, especially in 3D cultures (Xu et al., 2013). On the other hand, smaller pore sizes result in more intimate cell interactions, 3D cell aggregation, and a lower oxygen environment, which mimic the adverse environment within IVD and may correlate positively with IVD cell differentiation. Also, the biomaterials of contradictory reports are all different, which may also be an important reason. Therefore, though pore size has a significant influence on cellular proliferation and differentiation, it cannot decide the outcome alone, and multiple factors should be considered comprehensively.

### Fiber Structure

Fiber structure has a substantial impact on the morphology of implanted cells and may contribute to the directional differentiation of cells into specific cell types. Nanofibers, which mimic the natural fibrous microstructure of the NP and AF, have been shown to be efficient in chondrogenesis and IVD-like cell differentiation. MSCs cultured on 3D-nanofiber gels have been shown to have greater potential in differentiating into NP-like cells and producing cell-specific ECM components (Bian and Sun, 2015). The biomaterial Hydromatrix^®^ has been used to form 3D hydrogel nanofibers and has been shown to promote hMSC viability (Hansson et al., 2017), ECM production and the differentiation of hMSCs into IVD-like cells *in vitro*. Functional self-assembled peptide nanofibers have been shown to enhance the proliferation, chemotactic migration, and differentiation of BMSCs toward NPCs (Wu et al., 2016), thus restoring the NP-like ECM. Moreover, BMSCs cultured on peptides with fewer nanofibers and smaller nanofibers (19.24 ± 3.6 nm) have been shown to enhance cell proliferation and directional differentiation (Wu et al., 2016). Electrospun-aligned microfibers composed of PCL have been shown to exhibit high levels of cell colonization, alignment and AF-like ECM (Gluais et al., 2019). Scaffolds that form microfibers can also be used to regenerate AF defects *in vivo* and appear to not be cytotoxic to MSCs (Kang et al., 2017).

According to our knowledge, biomaterials of nanofibers are more frequently included in the attempt of regenerating IVD, especially NP tissues. Probably, nanofibers better mimic the native collagen (Yang et al., 2017). However, there are a lack of studies on the effects of microfibers on cell differentiation for IVD regeneration *in vivo* and *in vitro*. In other words, nanofibers have been widely accepted for IVD regeneration. The concern is what kinds of nanofibers can lead to better outcome. A hybrid scaffold generated from electrospun-aligned nanoyarn/three-dimensional porous nanofibrous promoted higher Collagen type II, sox-9 and aggrecan expression of BMSCs than individual application of electrospun-aligned nanoyarn or three-dimensional porous nanofibrous (Ma et al., 2018), which supports that the stem cells differentiation is largely dependent on the arrangement of nanofibers (Liu et al., 2015). In another study (Ma et al., 2018), BMSCs were cultured on the surface of porous nanofibrous, and they well infiltrated into the porous nanofibers and interacted with stagger nanoyarn on the bottom. Therefore, stem cells influenced by 3D structure and 2D aligned fibers may performed better tissue specific differentiation ability. Since IVD is a complex structure contained both crossed collagen within NP tissues and aligned collagen within AF tissues (Chuah et al., 2017), considering IVD as a integrity may better achieved stem cell therapy than only focusing on NP or AF regeneration.

### Biochemical Components

Because Collagen type I and II are the major components of IVD tissues, many studies have used Collagen type I or II as fundamental ingredients for the establishment of IVD biomaterials. Bioscaffolds composed of Collagen type II have been shown to have a high efficiency in promoting cell proliferation and the differentiation of ADSCs (Tao et al., 2016). When ADSCs were embedded in Collagen type II hydrogels compared with Collagen type I hydrogels, an upregulation of Collagen type IIA, collagen type IIB and aggrecan gene expressions and stronger alcian blue staining were observed, indicating a more pronounced differentiation of ADSCs to the cartilage lineage (Lu et al., 2008). Collagen type II alone has been shown to induce ADSC differentiation into NP-like cells (Zhou et al., 2018b), which was related to FoxA2 overexpression (Zhou et al., 2018a). Moreover, Collagen type II combined with other natural components and crosslinkers, such as chondroitin sulfate (Zhou et al., 2018c), hyaluronan (Calderon et al., 2010), genipin (Zhou et al., 2018b), N,N-(3-dimethylaminopropyl)-N′-ethylcarbodiimide and N-hydroxysuccinimide (EDAC/NHS) (Zhou et al., 2016), to modify its porosity and other micromechanical properties has also been shown to exert an improved effect on stem cell proliferation and directional differentiation.

Proteoglycans, including aggrecan, lumican, fibromodulin, decorin and perlecan, are other essential components of IVDs. The aggregation of aggrecan with hyaluronan forms a unique scaffold for hydration and resists compression within IVDs (Pratta et al., 2000). Materials consisting of chondroitin sulfate (Zhou et al., 2018c), chitosan (Zhu et al., 2017b), and HA have also been widely investigated. For example, the gelatin-HA methacrylate hydrogel has been shown to induce ADSC differentiation into NP-like cells, and the combination of the hydrogel and ADSCs improved the efficacy of *in vivo* repair (Chen et al., 2019b). Additionally, chitosan combined with alginate has been shown to improve the biocompatibility of alginate and commit BMSCs to the NP-like cell lineage with TGF-β3 (Naqvi and Buckley, 2015). In addition, a triple-interpenetrating network hydrogel composed of dextran, chitosan and teleostean has been shown to maintain MSC viability, promote cell proliferation and induce MSC differentiation toward a chondrogenic lineage (Smith et al., 2014). Interestingly, the combination of collagen and GAGs have been shown to more accurately mimic the microstructure of the NP and AF, making it a better strategy for IVD repair than the use of a single component. Spherical niche-like structures composed of Collagen type II and HA have been shown to be quite efficient in promoting rabbit ADSC proliferation and chondrogenic differentiation for IVD regeneration (Fontana et al., 2014).

Growth factors are promising molecules for directional differentiation toward a specific lineage. TGF-β1/3 has been shown to promote the differentiation of MSCs into NP-like cells and increase the levels of GAGs secreted by cells (Colombier et al., 2016). For the induction of differentiation toward AF cells, TGF-β3 promotes the differentiation of MSCs into AF-like cells, while TGF-β1 has no obvious differentiation-promoting effect (Blanquer et al., 2017). In addition to TGF-β, many molecules, such as insulin-like growth factor 1 (IGF-1), bone morphogenetic protein (BMP-7), and growth differentiation factor 5 (GDF-5), have been reported to affect the differentiation of stem cells into IVD cells (Feng et al., 2015). Thus, biomaterials with a growth factor delivery system could be an ideal and promising strategy for IVD tissue engineering. Gelatine microspheres for the delivery of TGF-β3 have been loaded onto a collagen/low-molecular weight HA semi-interpenetrating network to produce a novel structure that exerts excellent micromechanical properties and a greater capacity to induce NP-like cell differentiation than scaffolds without additional TGF-β3 (Tsaryk et al., 2015).

### External Features for Stem Cell Differentiation

Avascular, hypoxia, intermittent high pressure, and acid microenvironment is well known in native IVD tissues (Gonzalez Martinez et al., 2017). IVD cells have significantly different responses to various microenvironmental properties. Hypoxia can induce NP cells autophagy (Choi et al., 2016) and increase cell apoptosis (Choi et al., 2016). Excessive compression also downregulated NP cells proliferation and leads to IVD (Li et al., 2019). A kind of hydrophilic bile acid, tauroursodeoxycholic acid, could protect NP cells from excessive compression-induced death by reducing the apoptosis and necroptosis (Wang et al., 2018b). The incubation environment may also play a role in directional differentiation toward an IVD-specific phenotype (Xu et al., 2018).

Human MSCs cultured in a hydrogel under 3D or 2D conditions have been shown to have significantly different capacities of developing NP-like cells (Kumar et al., 2016). More specifically, 3D culture with mechanical stimulation has demonstrated the ability to support hMSC viability and differentiation toward an NP-like cell phenotype (Kumar et al., 2016). One explanation for the distinction might be the native microenvironmental properties of IVDs. Because immature NPCs typically reside within NP tissues, which are gel-like environments, 3D cultures that involve embedding cells into hydrogels could more accurately mimic the native structure of NP tissues and result in similar cellular attachment and interaction characteristics, which might contribute to ECM production by NPCs and the differentiation of stem cells into NP-like cell lineages. Moreover, rabbit ADSCs have been cultured on the surface of PECUU materials with a higher elastic modulus for 21 days, and the expression level of Collagen type I gradually increased, while the Collagen type II and GAG contents decreased, indicating the likelihood of AF cell differentiation (Zhu et al., 2016). These evidences suggested that 2D culture should allow stem cells to adapt to a native lamellar structure and promote AF-like cell differentiation. Moreover, to more accurately simulate native AF tissue, materials composed of multilayer structures should be developed, and stem cells should be embedded between the layers to investigate cell migration and differentiation capacities.

Compression, including axial compression and hydrostatic pressure, is a non-negligible factor that maintains the balance of matrix turnover in IVDs and influences MSC biological behavior. Cyclic compression applied by a perfusion bioreactor has been shown to promote chondrogenesis of hMSCs that were cocultured with human AF and NP cells (Tsai et al., 2014). Hydrostatic pressure also improves the chondrogenic differentiation ability of neonatal human dermal fibroblasts (nHDFs) with BMP-2 supplementation (Singh et al., 2011). Thus, compression, which is a fundamental characteristic of native IVDs, is a key regulator of cell differentiation. A study developed TGF-β3-loaded PLGANPs to form a 3D culture system for MSCs. And it reported that low magnitudes (5%) loaded on the 3D culture system significantly upregulated GAG and hydroxyproline content, and promoted Collagen type II, aggrecan, sox-9 expressions both in transcript and protein levels, in which activation of transient receptor potential vanilloid 4 (TRPV4) channel played an important role in transducing mechanical signal (Gan et al., 2018). A multicomponent spinal motion segment encapsulating MSCs was established as a 3D model for IVD. Cyclic compression improved fiber matrix arrangement, while directional differentiation of stem cells was not investigated in the study (Chik et al., 2015). Probably, cyclic compression may serve as a promoter to activate stem cells differentiation to native residual cells (NP cells or AF cells) to generate more ECM and cellular factors to adapt to the unfavorable environment. Therefore, compression should not be ignored when investigating the effects of IVD-regenerating materials on directional differentiation toward IVD cells.

Hypoxia has been reported to be an important factor that affects stem cell fate (Kim et al., 2019). IVDs are the largest non-vascularized structures in the human body, and the lack of vascularization creates a hypoxic microenvironment. Many studies investigations of biomaterials (Zhang et al., 2014; Thorpe et al., 2016) that have focused on directing stem cell differentiation toward IVD cells included hypoxia as a variable and reached a similar conclusion that hypoxia enhanced chondrogenic and NP-like cell differentiation, promoted the expression of tissue-related markers, and increased the amount of ECM. Moreover, a few studies have investigated the effect of hypoxia on AF and NP cells seeded on 3D scaffolds and found that hypoxia was effective in maintaining the NP cell phenotype but does not affect AF cells (Mwale et al., 2011; Feng et al., 2013). Therefore, future development of IVD biomaterials should take oxygen content into account and use chemicals (CoCl_2_ et al.) or other methods to induce cellular hypoxia response, so as to improve efficiency of inducing tissue specific differentiation, while minimize the unfavorable situation on cells survival induced by prolonged hypoxia (Rahar et al., 2018).

## Signaling Pathways Involved in Biomaterial-Induced Ivd-Like Cell Differentiation

**FIGURE 6:**
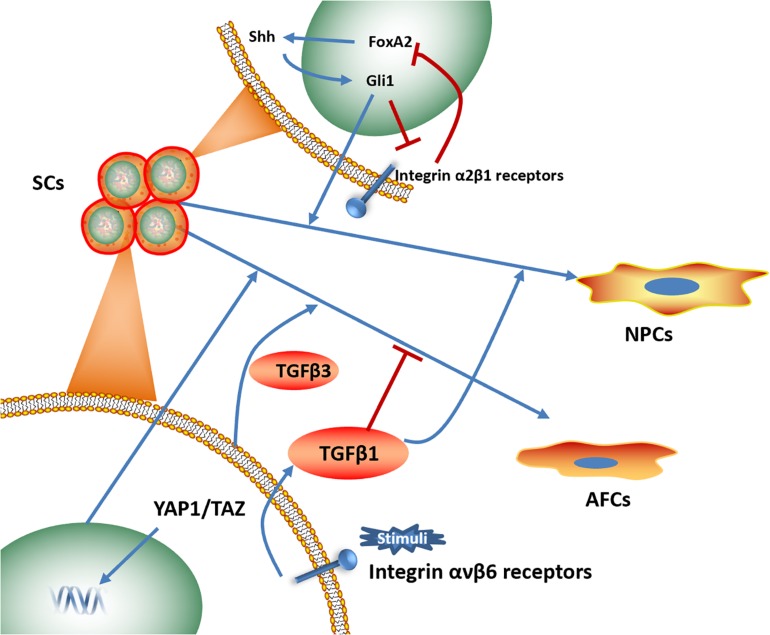
Signaling pathways associated with biomaterials-induced IVD cells directional differentiation (Dahia et al., 2012; Blanquer et al., 2017; Zhou et al., 2018a; Chen et al., 2019b; Chu et al., 2019). SCs, stem cells. NPCs, nucleus pulposus cells. AFCs, annulus fibrosus cells.

Numerous signaling pathways, including the Notch1, the Sonic Hedgehog (Shh) and NF-κB signaling pathways, have been shown to be related to IVD-like cell differentiation (Dahia et al., 2012; Cao et al., 2015; Lan et al., 2019). Identifying the key pathway that is most closely associated with biomaterials-induced IVD differentiation could provide novel perspectives for the future research and development of materials (Figure 6).

The integrin signaling pathway plays an essential role in NP interactions with ECM components, including collagen, fibronectin and laminin (Bridgen et al., 2013; Tran et al., 2014). Several studies have focused on the effects of the integrin signaling pathway on NP phenotype maintenance and NP-like cell differentiation. Increased expression levels of laminin receptors (integrin α3 and β4 subunits) was identified, and an NP-like phenotype was observed when human umbilical cord mesenchymal stromal cells (HUCMSCs) were cultured on lamini-1-rich Matrigel, suggesting the effect of the integrin signaling pathway on promoting NP-like cells differentiation (Chon et al., 2013). NP cells cultured on substrates coupled with the α3 integrin receptor peptides P4 and P678 and on the α2, α5, α6, β1 integrin-recognizing peptide AG10 have shown upregulated expression of aggrecan, N-cadherin, and Collagen types I and II (Bridgen et al., 2017). Moreover, a photocrosslinked GelHA hydrogel has been shown to induce NP-like cell differentiation for IVD repair by activating integrin αvβ6, and an integrin αvβ6-neutralizing antibody prevented this process *in vitro* (Chen et al., 2019b).

Similarly, Shh protein expression level has also been shown an increase when crosslinked scaffolds promoted ADSC proliferation and differentiation into NP-like cells, indicating that the activation of the Shh signaling pathway might play a regulatory role in directional differentiation (Zhou et al., 2018b). In addition, NP-like cell differentiation induced by Collagen type II is related to FoxA2 overexpression and can be rescued by a Shh signaling pathway inhibitor (Zhou et al., 2018a).

Yes-associated protein (YAP) has been shown to involve in differentiation of AFSCs. Outer AF phenotypic marker genes increased, when AFSCs were cultured on PECUU scaffolds with higher stiffness or greater fiber size, while YAP was translocated to the cell nucleus in AFSCs, suggesting the activation of YAP in outer AF-like cell differentiation (Chu et al., 2019).

Studies that have investigated signaling pathways involved in differentiation toward the IVD phenotype induced by biomaterials are relatively limited, and few studies have identified key regulators within the pathways that could efficiently influence the differentiation capacity of materials. Thus, future studies should combine biomaterials with pathway regulators to develop multifunctional materials for IVD regeneration.

## Limitations of Current Scaffold-Induced Ivd-Like Cell Differentiation and Outlook for Future Development

First, due to the significant differences in ingredients, processing methods and cell sources of several scaffold-based cellular delivery systems (Yoon et al., 2016), it is quite difficult to conclude which type of scaffold is optimal for the specific pathological disruption of IVDD. In addition, future studies should compare the effectiveness of different stem cell-carrying scaffolds across all pathophysiological stages of IVDD. Additional guidelines for the standardization of biomaterial manufacturing procedures and methodologies for the isolation, expansion and differentiation of various types of stem cells could substantially contribute to accelerating the clinical applications of materials for stem cell delivery. As for the injectable hydrogel material, no sealing of the annulus fibrosus after injection and hydrogel extrusion have been reported (Reitmaier et al., 2014). Thus, the sealant property of newly developed injectable hydrogels as AF/NP replacements for the biomechanical restoration and biological regeneration of the IVD tissues should be deeply tested to avoid material extrusion in clinical usage.

Moreover, although the number of suggested NP and AF phenotypic markers is gradually increasing as microarray studies are conducted, no final standards have been reached regarding the specificity of NP and AF markers (Pattappa et al., 2012; Wang et al., 2018a). These limitations greatly obstruct the specific detection of seeded cell differentiation along NP-/AF-like lineages. Thus, biomarkers of specific IVD cells continue to be identified for accurately evaluating the efficiency of the differentiation of MSCs loaded in biomaterials.

In addition, it has been reported that stiffness is a key biological signal for cell differentiation into IVD tissues. Moreover, native NP and AF have elastic moduli of 22 kPa (Bron et al., 2009) and 10–50 kPa (Cortes et al., 2013), respectively. The native modulus is much greater than the favorable modulus that is suitable for IVD-like cell differentiation (2 kPa). An excessive modulus leads to osteogenesis and calcification, which primarily degenerate IVD performance (Hsieh et al., 2016). Therefore, the modulus of biomaterials might be a major limitation for IVD-like cell differentiation. Thus, further studies should not only focus on the effect of the elastic modulus on generating native IVD tissues but also focus on other factors, such as the pore size, fiber diameter, growth factors and native and synthetic matrixes, to generate a favorable microenvironment for IVD-like cell differentiation.

Mesenchymal stem cells from different sources have been applied in IVD regeneration, including BMSCs, ADSCs, NP-derived stem cells, umbilical cord-derived MSCs and synovial-derived MSCs et al. One of the biggest barriers that hinder stem cells from achieving long-term benefits is the immunogenicity of either allogeneic or xenogeneic cell. Engineered MSCs are efficient approaches for better modification of immunogenicity. Cytomegalovirus US2/US3 gene was introduced into ADSCs to generate a ADSCs with decreased MHC I protein, thus reducing the activation of T-cells of recipients (Ren et al., 2012). Moreover, engineering MSCs, such as FoxA2-overexpression (Zhou et al., 2018a), is a potential approach to achieve specific differentiation without the supplement of growth factors (Liao, 2016), thus engineered MSCs may be ideal sources of cell delivery vehicles.

## Author Contributions

ZS and XQ contributed to the conception and design of this review article. YP and DH performed searches, analyses, and interpretations. SL and JL drafted the manuscript. YP substantially revised the manuscript. ZS and XQ gave final approval of the version to be submitted.

## Conflict of Interest

The authors declare that the research was conducted in the absence of any commercial or financial relationships that could be construed as a potential conflict of interest.
